# Irisin restrains neuroinflammation in mouse experimental autoimmune encephalomyelitis via regulating microglia activation

**DOI:** 10.3389/fphar.2025.1561939

**Published:** 2025-04-29

**Authors:** Qiu-Xia Zhang, Lin-Jie Zhang, Ning Zhao, Li Yang

**Affiliations:** Department of Neurology, Tianjin Neurological Institute, Tianjin Medical University General Hospital, Tianjin, China

**Keywords:** irisin, microglia, EAE, mechanism, therapy, neuroinflammation

## Abstract

**Introduction:**

Multiple sclerosis is a chronic autoimmune demyelinating disorder predominantly affecting the white matter of the central nervous system, with experimental autoimmune encephalomyelitis (EAE) serving as its classical animal model. Irisin, a glycosylated protein derived from the proteolytic cleavage of fibronectin type III domain-containing protein 5, plays a significant role in metabolic regulation and inflammatory modulation within the organism.

**Methods:**

In this study, we systematically investigated the therapeutic effects and underlying mechanism of Irisin on EAE and BV2 microglial cells through comprehensive methodologies including quantitative real-time polymerase chain reaction, immunofluorescence staining and western blot.

**Results:**

Irisin exerts neuroprotective effects in EAE mice, significantly ameliorating both clinical and pathological manifestations of the disease. Mechanistically, Irisin attenuated inflammatory response and reduced the number of microglia through NF-κBp65 signaling pathway.

**Conclusion:**

In conclusion, these results collectively suggest that Irisin alleviates EAE progression by suppressing microglia activation via the NF-κBp65 pathway, highlighting its potential as a promising therapeutic target for multiple sclerosis treatment.

## 1 Introduction

Multiple sclerosis (MS) is a chronic autoimmune disorder of the central nervous system (CNS), characterized by a spectrum of clinical manifestations including vision impairment, motor dysfunction, sensory disturbances, urinary and bowel incontinence, dizziness, and balance difficulty (Marcus, 2022). Epidemiological data indicate a global MS prevalence of 35.9 per 100,000 persons with an incidence rate of 2.1 per 100,000 persons/year. It is estimated that approximately 2.8 million people worldwide are affected by MS, with the average age of diagnosis being 32 years (Walton et al., 2020). This debilitating disease poses a significant threat to the quality of life and overall health of patients with MS, while also imposing a substantial socioeconomic burden on families and society. Therefore, there is an urgent need to elucidate the underlying pathogenic mechanisms of MS and to develop effective therapeutic interventions.

The pathogenesis of MS is multifactorial and complex, with current evidence suggesting a synergistic interplay of genetic predisposition, environmental factors, viral infections, and autoimmune dysregulation in driving disease onset and progression of MS (International Multiple Sclerosis Genetics C, 2019; i MCEasbue and iMSMS Consortium, 2022; Cox et al., 2021; Bjornevik et al., 2022; Wheeler et al., 2019; Reich et al., 2018). Experimental autoimmune encephalomyelitis (EAE) serves as a robust animal model for MS research, recapitulating key histopathological and immunological features of the human disease. Notably, many therapeutic agents currently used in MS treatment have been initially tested or validated using the EAE model (Dedoni et al., 2023). The pathological characteristics of MS/EAE include the infiltration of monocytes and lymphocytes into the CNS, activation of microglia, proliferation of astrocytes, and subsequent demyelination accompanied by axonal degeneration (Frischer et al., 2015; Charabati et al., 2023).

Microglia, the resident immune cells of the CNS, play a pivotal role in the pathological mechanisms in MS. These cells continuously surveil the CNS microenvironment, dynamically adapting their phenotype and function to the environmental cues, and rapidly respond to pathological changes with the CNS. Microglia are multifunctional cells including phagocytosis, antigen presentation, cytokine production, immune regulation and tissue repair (O'Loughlin et al., 2018; Dong and Yong, 2019; Sun et al., 2023). Microglia exhibit remarkable plasticity, capable of transitioning between proinflammatory phenotype and anti-inflammatory phenotype in MS/EAE. In their proinflammatory state, microglia release inflammatory mediators such as interleukin-6 (IL-6) and interferon-γ (IFN-γ), secrete chemokines including CCL2 and CCL3, and upregulate the expression of co-stimulatory molecules such as CD86, CD80 and CD40 (Chu et al., 2018). These activated microglia participate in antigen presentation, exacerbate neurotoxicity and contribute to neuronal and oligodendrocyte degeneration. Conversely, in their anti-inflammatory state, microglia secrete anti-inflammatory factors such as transforming growth factor-β (TGF-β), and facilitate the clearance of myelin debris through receptors including CD36, Fc receptors, complement receptor 3 (CR3) and Mer tyrosine kinase (Poppell et al., 2023). This reparative phenotype promotes neuronal survival and supports myelin regeneration (Lloyd and Miron, 2019).

Irisin was first identified by Bostrom et al., in 2012 as a glycosylated protein composing 112 amino acids, generated through the proteolytic cleavage of fibronectin type III domain containing protein 5 (FNDC5) (Timmons et al., 2012; Schumacher et al., 2013). Initially recognized for its physiological roles in inducing the browning of white adipocytes, enhancing glucose homeostasis, regulating lipid metabolism, and reducing insulin resistance (Waseem et al., 2022), Irisin has recently been implicated in the modulation of inflammatory diseases via the NF-κB and mitogen-activated protein kinase (MAPK) signaling pathways. *In vitro* studies have demonstrated that Irisin attenuates LPS-induced inflammation in RAW 264.7 macrophages by suppressing cytotoxicity and apoptosis via the MAPK pathway (Ma et al., 2023). Furthermore, in acute lung injury mice, Irisin has been shown to reduce inflammatory cells infiltration and the secretion of proinflammatory cytokines, while simultaneously promoting the polarization of proinflammatory macrophages toward an anti-inflammatory phenotype (Han et al., 2023). Notably, Irisin can cross the blood-brain barrier and exert anti-inflammatory effects within the CNS. Irisin effectively decreases the levels of tumour necrosis factor-α (TNF-α) and IL-6, inhibits the infiltration of microglia and monocytes into the CNS inflammatory lesions, and upregulates the expression of brain-derived neurotrophic factor, thereby conferring neuroprotection and regulatory benefits (Yu et al., 2022; Lourenco et al., 2019). Although research on the role of Irisin in the CNS remains limited, emerging evidence supports its anti-inflammatory properties in the CNS. Given these findings, we hypothesize that Irisin may hold therapeutic potential for MS, a classic inflammatory demyelinating disorder of the CNS.

In this study, we used the EAE mouse model and an *in vitro* inflammatory model using BV2 microglial cells to systematically evaluate the effects of Irisin in both *in vivo* and *in vitro* settings. Our findings demonstrated that Irisin administration significantly alleviated the clinical manifestations of EAE and reduced microglial activation in the CNS.

## 2 Materials and methods

### 2.1 Animals

The effects of biological sex in EAE vary amongst animal models and should not be widely extrapolated. In EAE models induced by C57BL/6 mice, the incidence rate is same in males and females (McCombe Pamela and Judith, 2022; Yoshinobu et al., 2002a). Considering that multiple sclerosis is more prevalent in young women than men, we ultimately chose 6–8 weeks female mice. Female, 6–8-week-old C57BL/6 mice (18–20 g) were purchased from Vitong Lihua Laboratory Animal Technology (Beijing, China). All animals were housed in specific pathogen free laboratories under temperatures (22°C ± 2°C) with a 12-h light/dark cycle and they were allowed free access to food and water in the Laboratory Animal Center. There were six mice per cage during the experiment. All efforts were made to minimize the number of animals used and any suffering they might experience. There were two points that we need to pay attention to in order to alleviate animal suffering. The one was reduction. We improved experimental design and techniques, and minimized the number of mice as much as possible. The other was refinement. By improving experimental conditions and optimizing experimental techniques, we can avoid or alleviate the pain caused to mice including selecting appropriate anesthetics and euthanasia methods. The experimental protocols were approved by the Ethics Committee of Tianjin Medical University General Hospital (grant no. IRB2024-DWFL-309).

### 2.2 Induction and grouping of EAE

For EAE induction, C57BL/6 mice were injected subcutaneously with 200 μg of myelin oligodendrocyte glycoprotein (MOG) 35–55 emulsified in complete Freund’s adjuvant containing heat-inactivated *Mycobacterium tuberculosis* (Difco Laboratories, United States) at two sites, with 100μL/site. Pertussis toxin (250ng, List Biological Laboratories, United States) was injected intraperitoneally on days 0 and 2. Mice were observed and weighted daily after immunization. And Table 1 was the grading criteria as mentioned earlier (Zhang et al., 2021).

**TABLE 1:** Grading criteria.

Grade	Clinical manifestation
0.0	no clinical symptoms
1.0	tail paralysis
2.0	uncoordinated gait and slight paralysis of the hind limbs
3.0	paralysis of both hind limbs
4.0	forelimb paralysis
5.0	death

**FIGURE 1:**
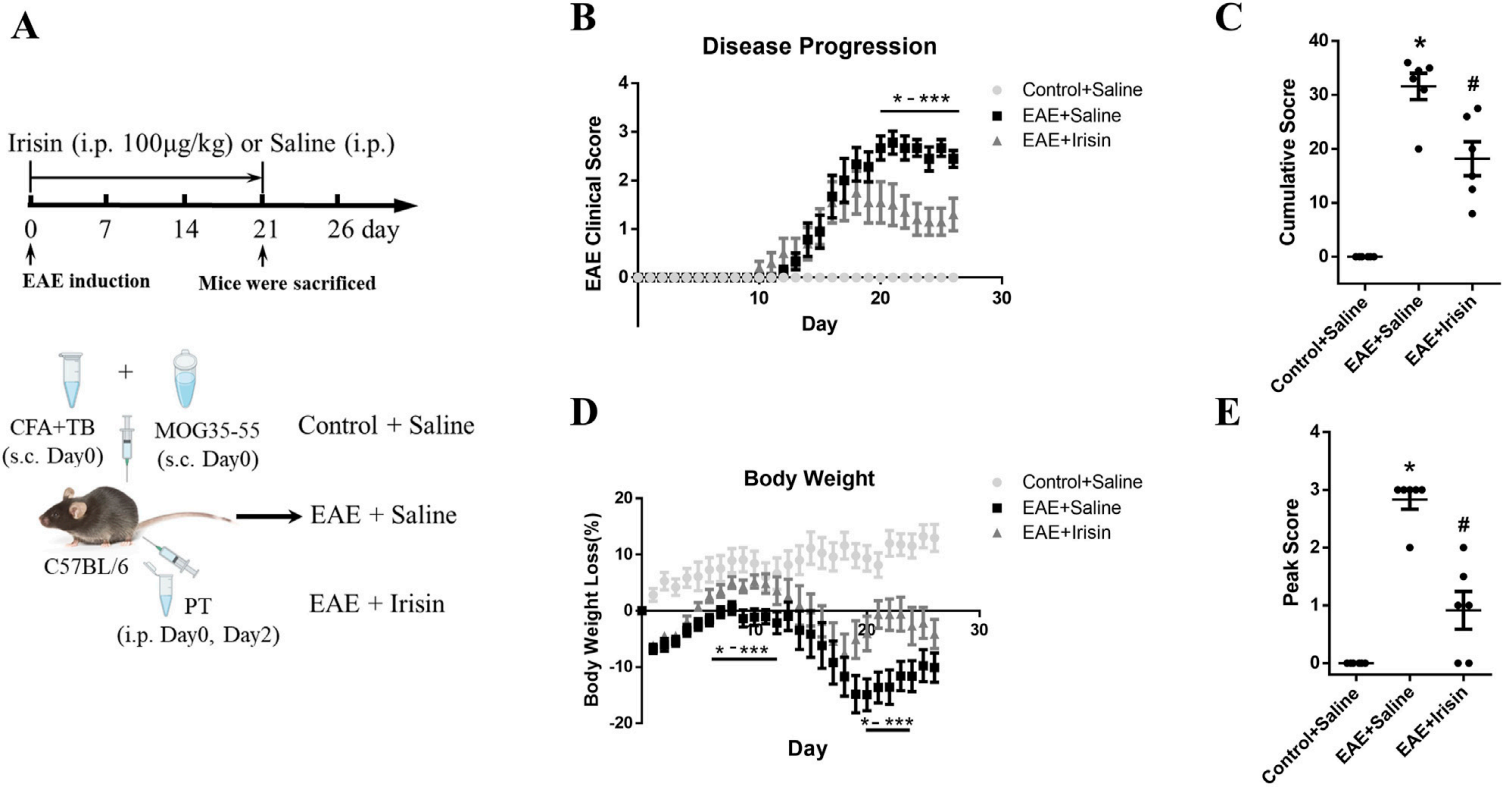
Irisin improved clinical scores and weight loss in EAE mice. **(A)** The experimental design applied *in vivo*. **(B)** Irisin could improve the clinical scores of EAE mice. On the 20th to 26th day after immunization, the clinical scores in the EAE + Irisin group were significantly reduced compared to the EAE + Saline group. **(C)** Cumulative scores were compared between the groups of Control + Saline, EAE + Saline and EAE + Irisin. F (2, 15) = 47.75, *P < 0.0001, #P = 0.013. **(D)** Irisin could improve the weight loss of EAE mice. Weight changes were recorded and compared between the three groups. Compared to the EAE + Saline group, the EAE + Irisin group significantly improved body weight on the 5th to 12th and 19th to 25th day after immunization. **(E)** Peak scores were compared between the groups of Control + Saline, EAE + Saline and EAE + Irisin. F (2, 15) = 46.55, *P < 0.0001, #P = 0.040. There were six mice per group and data were Mean ± SD.

Animals were randomly assigned to three groups, namely, blank control group (Control + Saline), model control group (EAE + Saline) and Irisin intervention group (EAE + Irisin). Irisin was purchased from AdipoGen Life Sciences. There were six mice in each group. From the 1st to 21st days after immunization, the Irisin intervention group mice were given daily intraperitoneal injection of Irisin (100 μg/kg). Simultaneously, the blank control group and model control group mice were given daily intraperitoneal injection of an equal amount of saline. We observed the mice daily after immunization and weighed them until the 26th day. As mentioned earlier, the peak of disease refers to the 17th to 21st days after immunization (Zhang et al., 2021). On the 21st day after immunization, another group of mice were euthanized to obtain lumbar spinal cord, and the histopathology, immunofluorescence, western blotting and qPCR were conducted (Figure 1A).

### 2.3 Hematoxylin-eosin and luxol fast blue staining

Lumbar spinal cords of mice were fixed with 4% formaldehyde for 24h, embedded in paraffin, and sliced into 8 μm sections. After deparaffinization and hydration, hematoxylin-eosin (HE) staining was performed with hematoxylin for 2 min and eosin for 2 min. For luxol fast blue (LFB) staining, the hydrated paraffin sections were stored in LFB solution at 60°C for 2 h. After washed in ethanol and distilled water, the slices were kept in saturated lithium carbonate solution 0.05% for 10s. Repeat the above operation until the observer could clearly distinguish between grey matter and white matter. Three slides from each spinal cord were analyzed under a fluorescence microscope (Olympus, Japan). Semiquantitative histological evaluation for inflammation and demyelination were performed (Akimasa et al., 2015; Yoshinobu et al., 2002b). The scoring criteria for HE staining were as follows: 0, normal; 1, cellular infiltration only in the perivascular areas and meninges; 2, mild cellular infiltration (less than one-third part of the white matter is infiltrated with inflammatory cells); 3, moderate cellular infiltration (more than one-third part of the white matter is infiltrated with inflammatory cells); and 4, infiltration of inflammatory cells was observed in the whole white matter. LFB staining was evaluated according to the following scoring system: 0, normal; 1, rare demyelination; 2, a few areas of demyelination; 3, one to two large areas of demyelination; and 4, extensive demyelination. To avoid bias, the experiments were conducted in a double-blind evaluation. Specifically, two independent observers (who were blinded to the grouping of the experiment) scored according to the same criteria.

### 2.4 Quantitative real-time polymerase chain reaction (qRT-PCR)

Lumbar spinal cord of mice and BV2 cells were acquired for RNA extraction. Total RNA was extracted by TRIzol reagent (Invitrogen, United States). And then the total RNA was reverse-transcribed into cDNA using TransScript^®^ Fly first-strand cDNA synthesis supermix. The amount of RNA and cDNA was 1 μg and 0.2μg, respectively. The SYBR Green Master Mix was further used to measure the mRNA levels. The specific primer was shown in Table 2. RT-PCR was performed on the Real-Time PCR Detection System (Bio-Rad). The 2^-△△Ct^ method was used to measure relative expression levels of each gene. GAPDH was used as an internal reference for genes.

**TABLE 2:** Primer sequences.

Gene	Forward	Reverse
IL-6	CCG​GAG​AGG​AGA​CTT​CAC​AG	TCT​GCA​AGT​GCA​TCA​TCG​TT
IL-1β	GCT​GAA​AGC​TCT​CCA​CCT​CA	AGG​CCA​CAG​GTA​TTT​TGT​CG
TNF-α	ATG​TCT​CAG​CCT​CTT​CTC​ATT​C	GCT​TGT​CAC​TCG​AAT​TTT​GAG​A
iNOS	GAG​CTG​GGC​TGT​ACA​AAC​CTT	CAT​TGG​AAG​TGA​AGC​GTT​TCG
CD86	TTG​TGT​GTG​TTC​TGG​AAA​CGG​AG	AAC​TTA​GAG​GCT​GTG​TTG​CTG​GG
GAPDH	TGT​GAT​GGG​TGT​GAA​CCA​CGA​GAA	CAT​GAG​CCC​TTC​CAC​AAT​GCC​AAA

### 2.5 Immunofluorescence

Lumbar spinal cords of mice were fixed with 4% formaldehyde overnight, embedded in paraffin, sliced, hydrated, permeabilized and then blocked. The slides were cultured with primary rabbit anti-mouse antibodies against ionized calcium binding adapter molecule 1(Iba1) (1:500, Wako) at 4°C overnight and with secondary goat anti-rabbit antibody (1:500, Invitrogen) for 1 h at 37°C. Three slides from each spinal cord were analyzed under a fluorescence microscope (Olympus, Japan).

### 2.6 Western blot

Western blot was performed as previously described (Zhang QX. et al., 2024). The total protein was extracted from spinal cords of mice and BV2 cells using RIPA buffer (Solarbio Science and Technology) and 20 μg protein was loaded onto gels for western blot. After electrophoresis, membrane transfer and blocking, the membranes loaded with protein were incubated with rabbit anti-p-NF-κBp65 (1:1000, Cell Signaling Technology), rabbit anti-NF-κBp65 (1:1000, Cell Signaling Technology) and rabbit anti-β-actin (1:1000, Cell Signaling Technology) at 4°C in the dark overnight. And then the membranes were incubated in goat anti-rabbit secondary antibody (1:10,000, Zhongshan Jinqiao Biotechnology) labelled with horseradish peroxidase for 1 h at 37°C. We used StarRuler Color Prestained Protein Marker (10–270kDa, Genstar) as molecular mass marker. The protein-specific signals were detected using a Bio-Rad Gel Doc Imager (Bio-Rad). The antigen-antibody complexes were detected using ECL reagent (Merck Millipore) and quantified by ImageJ software. The amount of targeted proteins was normalized relative to β-actin.

### 2.7 Cell culture

BV2 cells were cultured in DMEM medium supplemented with 10% foetal bovine serum and 1% penicillin–streptomycin at 37°C in 5% CO2 under constant temperature and humidity. BV2 cells were seeded at a density of 2 × 10^5^/per well into the 6-wells plates. Previous studies have shown that BV2 cells are stimulated with lipopolysaccharide (LPS) and IFN-γ (Pan et al., 2023; Fan et al., 2023; Velagapudi et al., 2014). In the study, we added LPS (200 ng/mL) and IFN-γ (20 ng/mL) to the medium to construct inflammatory model of BV2 cells. The cell experiment was divided into three groups: control group (BV2), inflammatory model group (BV2 + LPS + IFN-γ) and Irisin intervention group (BV2 + LPS + IFN-γ + Irisin). Based on the cell viability assay, we used 200 ng/mL Irisin for cell experiments. Cells were collected from each group for experiments such as qRT-PCR and western blot after 24 h (Figure 6A). There were four per group and the experiment was performed three times.

### 2.8 Cell viability assay

The cell counting kit-8 (CCK-8, YEASN) was used for determination of cell viability. In brief, BV2 cells were seeded and treated with various concentrations of Irisin (0, 12.5, 25, 50, 100 and 200 ng/mL) (Choi et al., 2024; Qiao et al., 2018; Zheng et al., 2018; Ye et al., 2020) at a density of 1 × 10^4^/per well into the 96-wells plates for 24 h. The medium was then removed and the cells were incubated with 10 μL CCK-8 solution for 1 h and optical density was measured at 450 nm.

### 2.9 Statistical analysis

Statistical analyses were performed using Statistical Package for the Social Sciences (SPSS 22.0), and graphs were created using GraphPad Prism version 6.0 software. Data from results were statistically analyzed using one-way analysis of variance (ANOVA) followed by Dunnett’s multiple comparisons test. P-values of <0.05 were considered statistically significant. *P < 0.05, EAE + Saline vs. Control + Saline; #P < 0.05, EAE + Irisin vs. EAE + Saline; P < 0.05, BV2 + LPS + IFN-γ vs. BV2; #P < 0.05, BV2 + LPS + IFN-γ + Irisin vs. BV2 + LPS + IFN-γ.

## 3 Results

### 3.1 Irisin improved clinical scores and weight loss in EAE mice

To investigate the effect of Irisin on EAE mice, we divided the mice into three groups: blank control group (Control + Saline), model control group (EAE + Saline), and Irisin intervention group (EAE + Irisin). After the induction of EAE, we recorded behavioural and neurologic scores of mice and weighed their weight every day. The experimental results were as follows: the group of Control + Saline did not develop any disease, and the clinical scores were all zero. The group of EAE + Saline and EAE + Irisin gradually developed disease from 10th to 14th days after immunization, and there was no significant statistical difference in the onset time between the two groups. From the 17th to 21st days after immunization, the group of EAE + Saline and EAE + Irisin gradually reached the peak of disease. Compared with the group of EAE + Saline, the group of EAE + Irisin showed a significant reduction in clinical scores on the 20th to 26th days after immunization (P < 0.05, Figure 1B). We further compared cumulative and peak scores of mice in the three groups to assess the most severe symptoms that the animals experienced. The group of EAE + Saline had a cumulative score of 31.50 ± 5.42, which was significantly higher than the group of EAE + Irisin (20.14 ± 8.11) (P = 0.013, Figure 1C). As shown in Figure 1E, peak scores followed a similar trend, where the EAE + Saline mice peaked at 2.67 ± 0.71, while the EAE + Irisin mice were 1.55 ± 1.34 (P = 0.040). The weight of Control + Saline mice remained stable and showed an upward trend, while the weight of EAE + Saline and EAE + Irisin mice showed a gradual decrease followed by a slow recovery process. On the 5th to 12th days and the 19th to 25th days after immunization, the group of EAE + Irisin gained weight faster than the group of EAE + Saline (P < 0.05, Figure 1D). These above results suggested that Irisin could alleviate the clinical symptoms of EAE mice and might have a protective effect on EAE mice.

### 3.2 Irisin reduced inflammatory cells infiltration and myelin loss in the spinal cord of EAE mice

**FIGURE 2:**
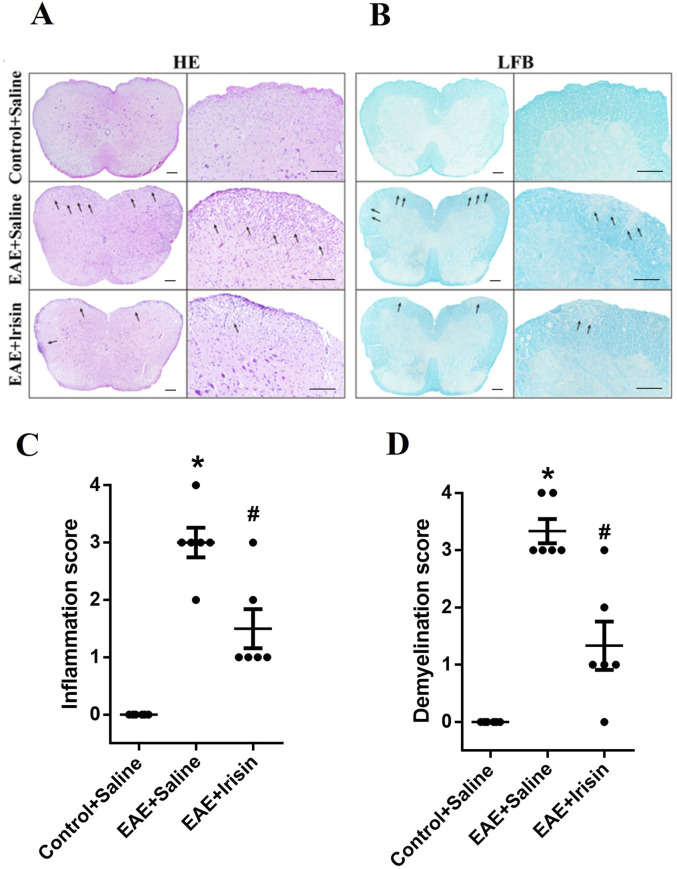
Irisin reduced inflammatory cells infiltration and myelin loss in the spinal cord of EAE mice. **(A)** Hematoxylin-eosin staining (HE) staining of the lumbar spinal cord. **(B)** Luxol fast blue (LFB) staining of the lumbar spinal cord. **(C)** Inflammatory scores of HE staining in the lumbar spinal cord between the groups of Control + Saline, EAE + Saline and EAE + Irisin. F (2, 15) = 35.24, *P < 0.0001, #P = 0.011. **(D)** Demyelination scores of LFB staining in the lumbar spinal cord between the groups of Control + Saline, EAE + Saline and EAE + Irisin. F (2, 15) = 34.59, *P < 0.0001, #P = 0.004. There were six mice per group and data were Mean ± SD. Bar = 200 μm.

We further evaluated the degree of inflammatory cells infiltration and demyelination in the spinal cord tissue sections by HE staining and LFB staining, respectively. The experimental results were shown in Figure 2. The Control + Saline group had almost no inflammatory cells infiltration (0.0 ± 0.0), the EAE + Saline group had a large amount of inflammatory cells infiltration (2.89 ± 0.31), and the EAE + Irisin group had a small amount of inflammatory cells infiltration (1.67 ± 0.29). It was found that the degree of inflammatory cells infiltration in the EAE + Irisin group was significantly reduced compared to the EAE + Saline group, and the difference was statistically significant (P = 0.011, Figures 2A,C). The scores of myelin loss in the groups of Control + Saline, EAE + Saline and EAE + Irisin were 0.0 ± 0.0, 3.11 ± 0.31, and 1.56 ± 0.34, respectively. Compared with the EAE + Saline group, the scores of demyelination in the EAE + Irisin group were significantly reduced (P = 0.004, Figures 2B,D). Consistently, these results indicated that Irisin could alleviate the inflammatory response and reduce myelin loss in the spinal cord of EAE mice.

### 3.3 Irisin could decrease the expression of inflammatory factors and the number of microglia in the spinal cord of EAE mice

**FIGURE 3:**
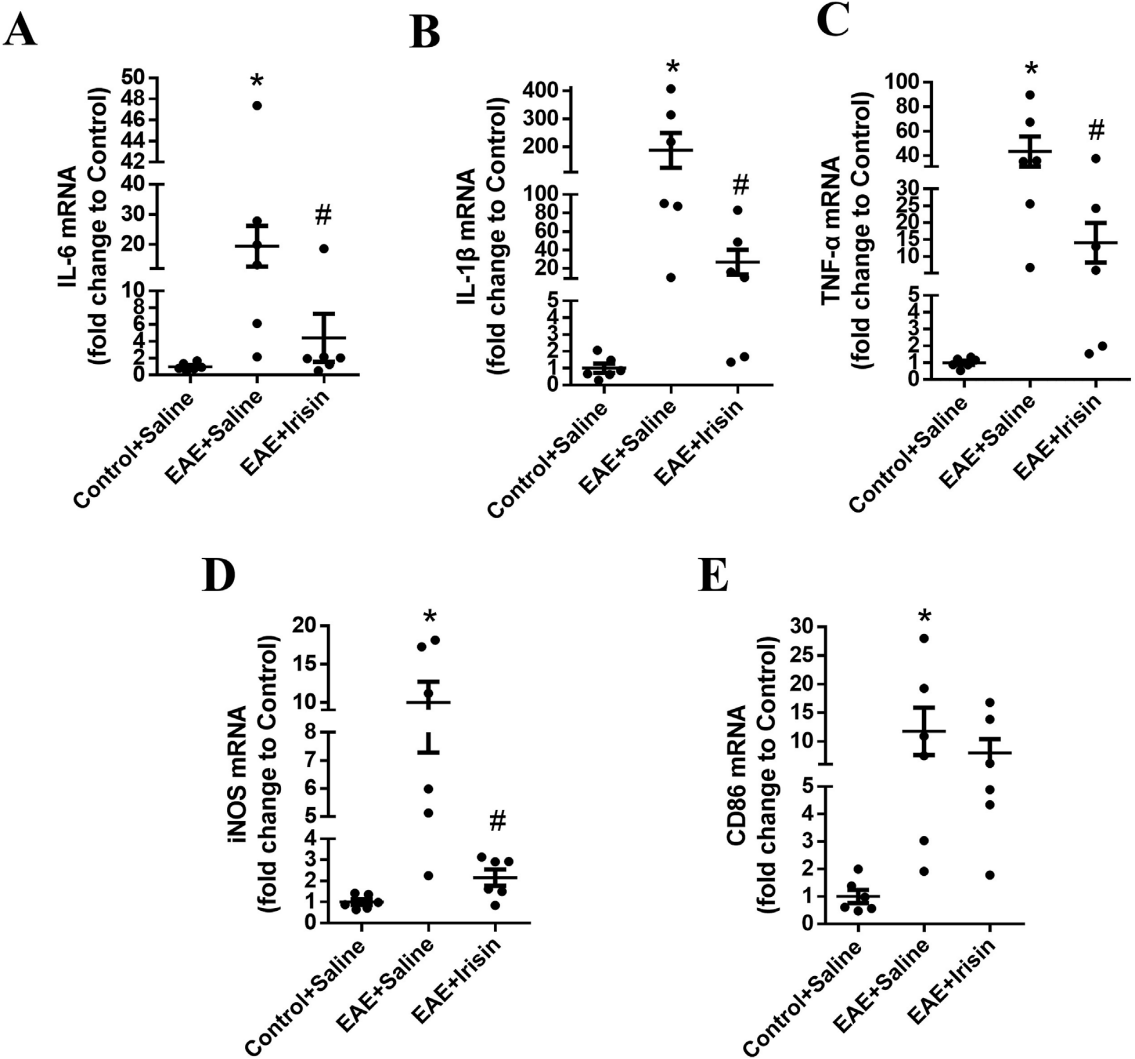
Irisin could decrease the expression of inflammatory factors in the spinal cord of EAE mice. The mRNA levels of IL-6 **(A)**, IL-1β **(B)**, tumor necrosis factor-α (TNF-α) **(C)**, inducible nitric oxide synthase (iNOS) **(D)**, and CD86 **(E)** in the spinal cord of the groups of Control + Saline, EAE + Saline and EAE + Irisin. F (2, 15) = 5.387, F (2, 15) = 7.600, F (2, 15) = 7.656, F (2, 15) = 9.550, F (2, 15) = 3.884; *P = 0.021, *P = 0.013, *P = 0.006, *P = 0.008, *P = 0.026; #P = 0.041, #P = 0.029, #P = 0.044, #P = 0.017, #P = 0.447. There were six mice per group and data were Mean ± SD.

To investigated the effect of Irisin on the expression of inflammatory factors of EAE mice, qPCR was used to detect the mRNA levels of IL-6, IL-1β and TNF-α in the spinal cord of mice during the peak period of disease. As shown in Figures 3A–C, the IL-6, IL-1β and TNF-α mRNA levels in the spinal cord of the EAE + Saline group significantly increased compared with the Control + Saline group (P = 0.021, P = 0.013 and P = 0.006, respectively); the IL-6, IL-1β and TNF-α mRNA levels in the spinal cord of the EAE + Irisin group significantly decreased compared with the EAE + Saline group (P = 0.041, P = 0.029 and P = 0.044, respectively). The above results suggested that Irisin had an anti-inflammatory effect.

**FIGURE 4:**
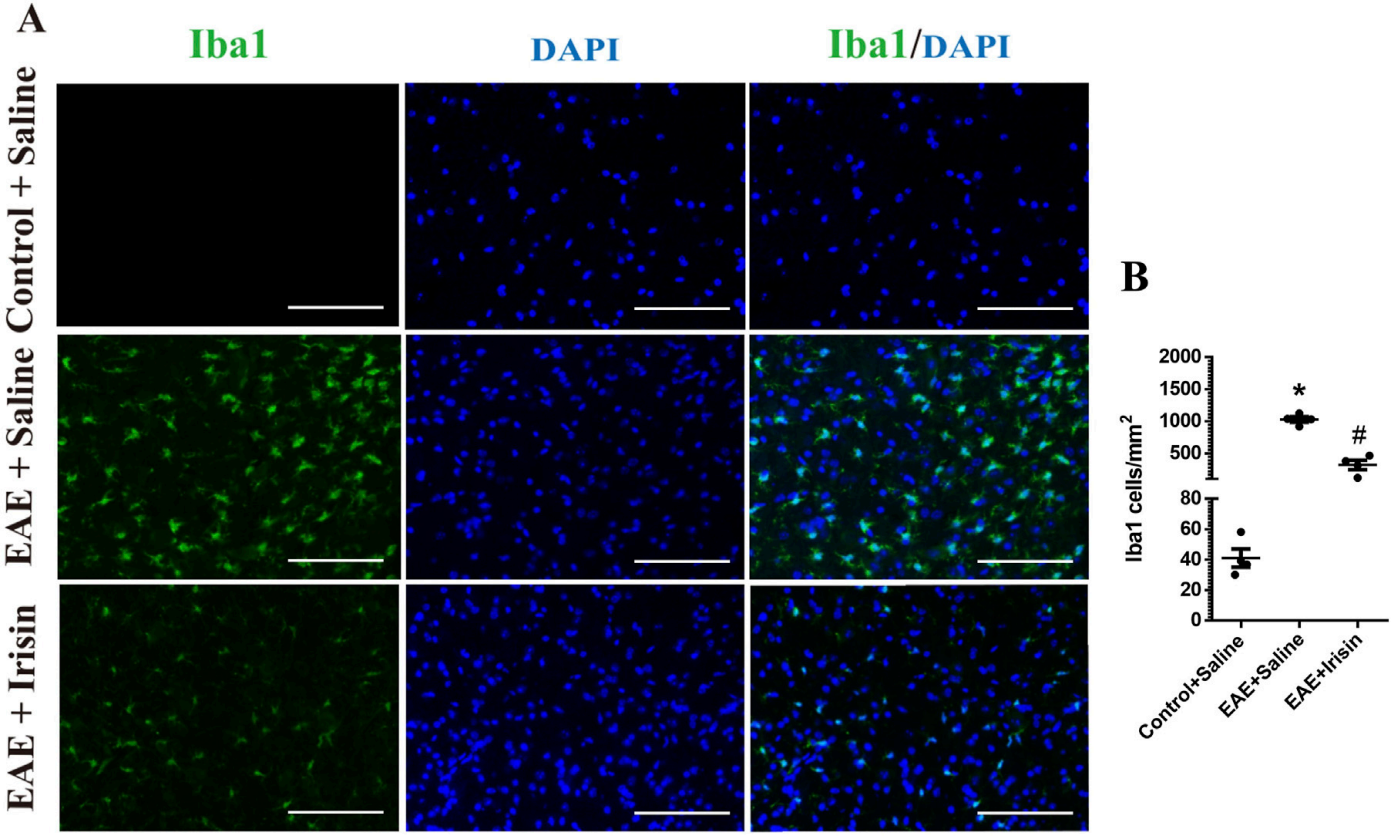
Irisin reduced the number of microglia in the lumbar spinal cord of EAE mice. **(A)** Immunofluorescence staining of microglia in the lumbar spinal cord. **(B)** The number of microglia in the lumbar spinal cord between the groups of Control + Saline, EAE + Saline and EAE + Irisin. Iba1, ionized calcium binding adaptor molecule 1. F (2, 9) = 107.8, *P < 0.0001, #P = 0.0002. There were four mice per group. Bar = 200 μm.

In addition, immunofluorescence staining was used to detect the expression of microglia in spinal cord of the three groups. The number of microglia in spinal cord of mice in the EAE + Saline group (1027.0 ± 41.8 Iba1 cells/mm^2^) were significantly higher than the Control + Saline group (41.1 ± 6.0 Iba1 cells/mm^2^) (P < 0.0001, Figure 4); the number of microglia in spinal cord of mice in the EAE + Irisin group (319.6 ± 73.5 Iba1 cells/mm^2^) were significantly lower than that in the EAE + Saline group (P = 0.0002, Figure 4). After activation, microglia could polarize into an inflammatory phenotype expressing iNOS and CD86. As shown in Figures 3D,E, the iNOS and CD86 mRNA levels in the spinal cord of the EAE + Saline group mice were significantly increased compared with the Control + Saline group (P = 0.008 and P = 0.026, respectively); Compared with the EAE + Saline group, the iNOS mRNA levels in the EAE + Irisin group significantly decreased (P = 0.017), the CD86 mRNA levels in EAE + Irisin group showed a downward trend, but the difference was not statistically significant (P = 0.447). In conclusion, Irisin could significantly reduce the number of microglia in spinal cord of EAE mice.

### 3.4 Irisin inhibited NF-κBp65 pathway in the spinal cord of EAE mice

**FIGURE 5:**
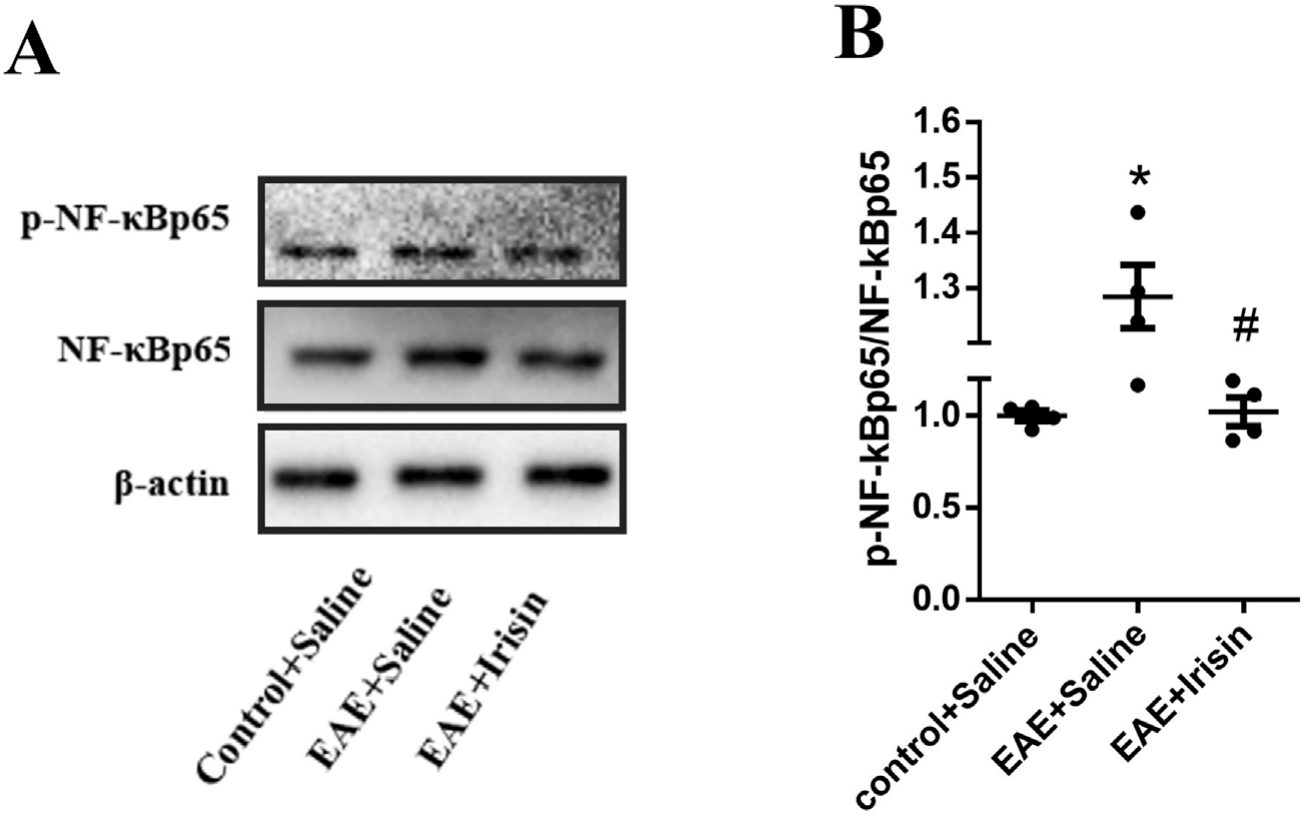
Irisin inhibited NF-κBp65 pathway in the spinal cord of EAE mice. **(A)** Detection the p-NF-κBp65/NF-κBp65 in the spinal cord of mice by western blot (WB). **(B)** Comparison the protein levels of p-NF-κBp65/NF-κBp65 in the spinal cord of mice between the groups of Control + Saline, EAE + Saline and EAE + Irisin. F (2, 9) = 7.423, *P = 0.004, #P = 0.035. There were four mice per group.

The above results showed that Irisin could alleviate the clinical symptoms of EAE mice, reduce inflammatory cells infiltration and myelin loss, and decrease the expression of inflammatory factors. The specific mechanism of Irisin to protect EAE mice remained unclear, so we detected p-NF-κBp65/NF-κBp65 in three groups by western blot. Compared with the Control + Saline group, the levels of p-NF-κBp65/NF-κBp65 in the spinal cord of the EAE + Saline group significantly increased (P = 0.004, Figure 5); The levels of p-NF-κBp65/NF-κBp65 in the spinal cord of the EAE + Irisin group significantly decreased compared with the EAE + Saline group (P = 0.035, Figure 5). Therefore, we concluded that Irisin might protect EAE mice by inhibiting the NF-κBp65 pathway.

### 3.5 The viability of BV2 cells after Irisin intervention

**FIGURE 6:**
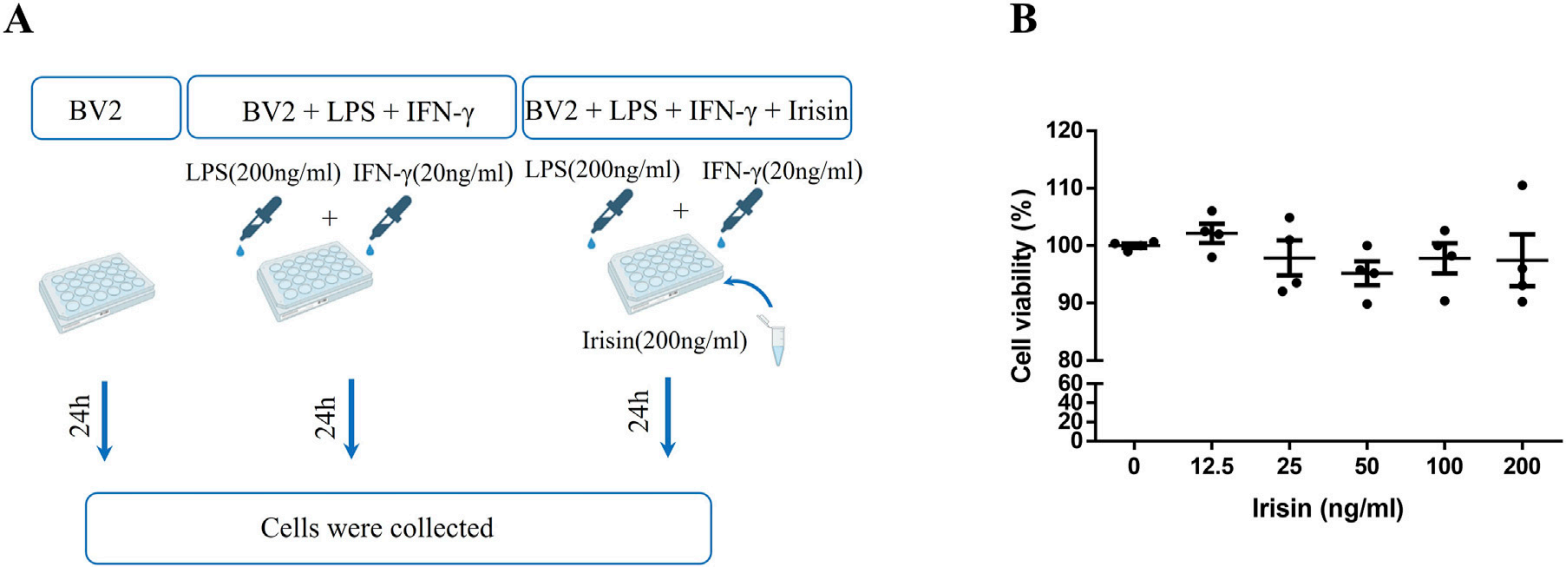
**(A)** The experimental design applied *in vitro*. **(B)** The change of viability of BV2 cells after Irisin intervention. Detection the viability of BV2 cells after Irisin intervention (0–200 ng/mL) by cell counting kit-8. F (5, 18) = 0.772, P = 0.583. There were four per group.

In order to clarify the effect of Irisin on the viability of BV2 cells and explore the appropriate concentration of Irisin for subsequent cell experiments, we used CCK-8 assay to detect the viability of BV2 cells after Irisin intervention (0, 12.5, 25, 50, 100 and 200 ng/mL). Compared with the group without Irisin, the activity of BV2 cells after Irisin intervention had no significant changes and the difference was not statistically significant (P > 0.05, Figure 6B). And we chose 200 ng/mL Irisin in subsequent cell experiments.

### 3.6 Irisin reduced the levels of inflammatory factors of BV2 cells

**FIGURE 7:**
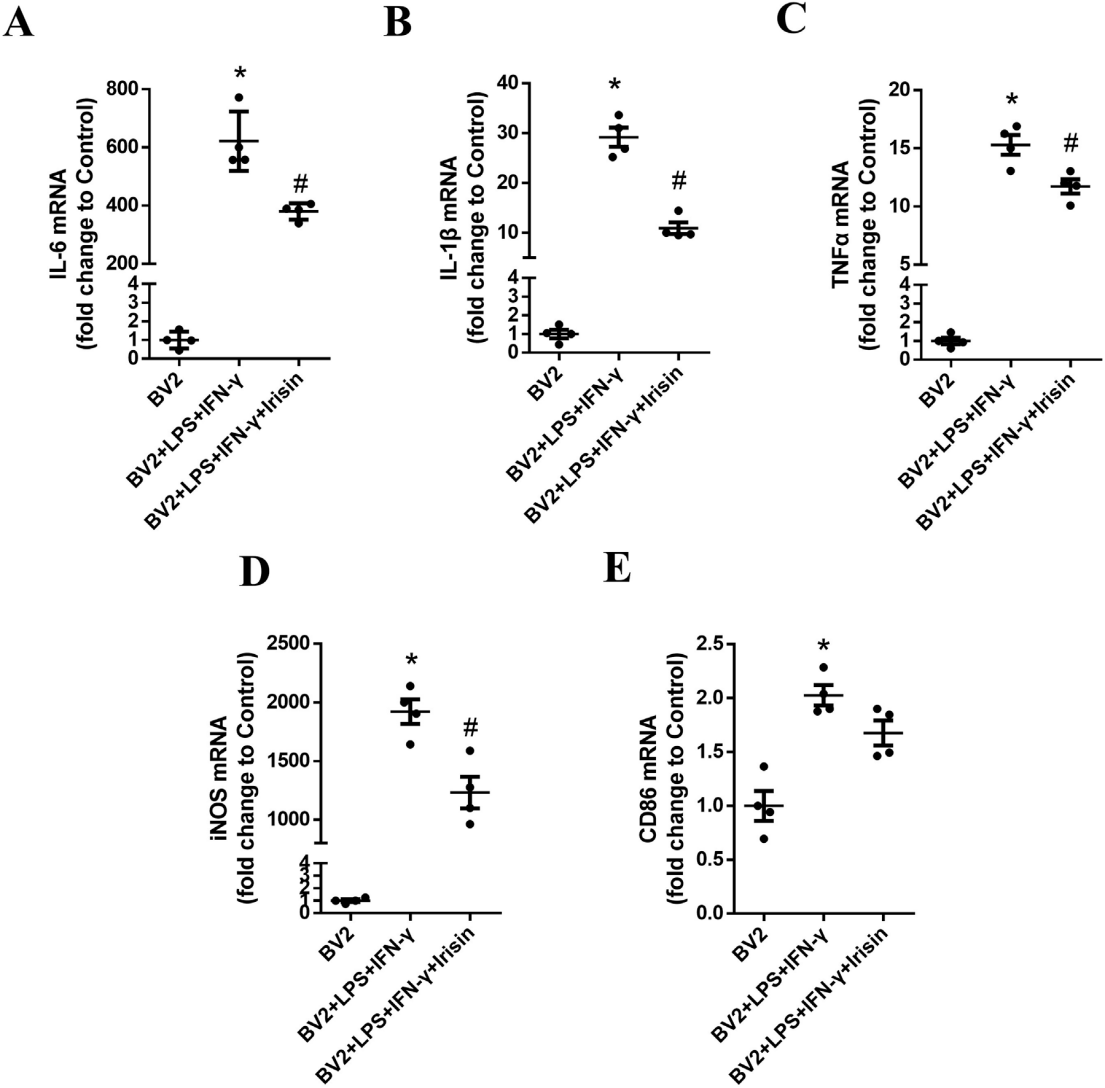
Irisin reduced the levels of inflammatory factors of BV2 cells. **(A)** The mRNA levels of IL-6 in the groups of BV2, BV2 + LPS + IFN-γ and BV2 + LPS + IFN-γ + Irisin. **(B)** The mRNA levels of IL-1β in the three groups. **(C)** The mRNA levels of TNF-α in the three groups. **(D)** The mRNA levels of iNOS in the three groups. **(E)** The mRNA levels of CD86 in the three groups. F (2, 9) = 104.4, F (2, 9) = 120.3, F (2, 9) = 148.2, F (2, 9) = 96.81, F (2, 9) = 19.82; *P = 0.0009, *P = 0.0004, *P = 0.0003, *P = 0.0002, *P = 0.009; #P = 0.028, #P = 0.005, #P = 0.031, #P = 0.032, #P = 0.053. There were four per group.

As shown in Figure 7, the mRNA levels of IL-6, IL-1β, TNF-α, iNOS and CD86 in the inflammatory model group significantly increased compared with the control group (P = 0.0009, P = 0.0004, P = 0.0003, P = 0.0002 and P = 0.009, respectively). Compared with the inflammatory model group, the mRNA levels of IL-6, IL-1β, TNF-α and iNOS in the Irisin intervention group significantly decreased and the difference was statistically significant (P = 0.028, P = 0.005, P = 0.031 and P = 0.032, respectively); the CD86 mRNA levels in the Irisin intervention group showed a decreasing trend, but the difference was not statistically significant (P = 0.053). Consistent with the vivo experimental results, Irisin could effectively inhibit the activation of BV2 cells and alleviate inflammatory reactions.

### 3.7 Irisin might inhibit the activation of BV2 cells through NF-κBp65 pathway

**FIGURE 8:**
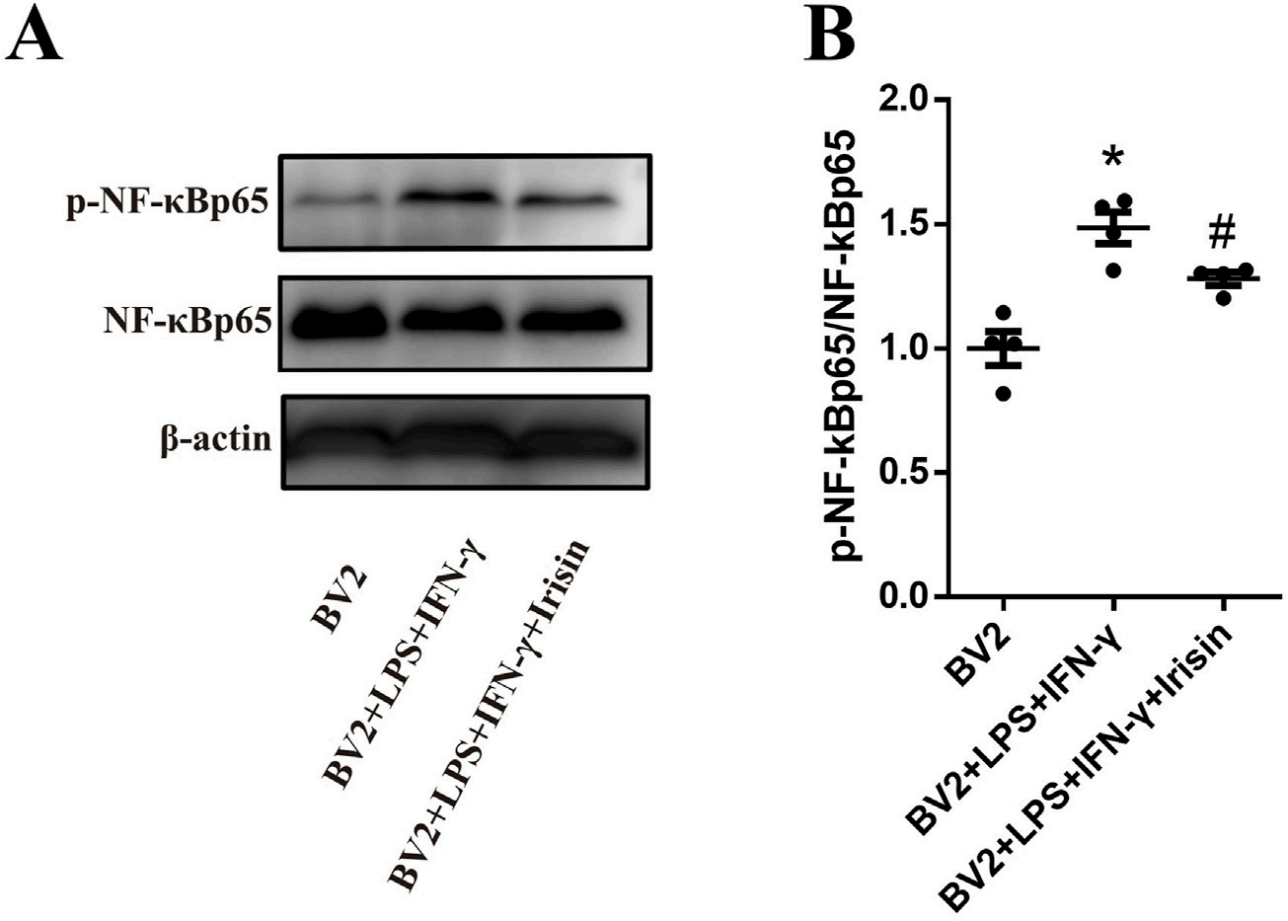
Irisin inhibited the activation of BV2 cells through NF-κBp65 pathway. **(A)** Detection the p-NF-κBp65/NF-κBp65 in the groups of BV2, BV2 + LPS + IFN-γ and BV2 + LPS + IFN-γ + Irisin by WB. **(B)** Comparison the protein levels of p-NF-κBp65/NF-κBp65 between the three groups. F (2, 9) = 19.12, *P < 0.0001, #P = 0.041. There were four per group.

We explored the signal pathway of Irisin to make anti-inflammatory effect on BV2 cells. As shown in Figure 8, the protein levels of p-NF-κBp65/NF-κBp65 in the inflammatory model group significantly increased compared to the control group (P < 0.0001); the protein levels of p-NF-κBp65/NF-κBp65 in the Irisin intervention group significantly decreased compared with the inflammatory model group (P = 0.041). The above results suggested that Irisin might inhibit BV2 cells activation through NF-κBp65 pathway.

## 4 Discussion

MS is a complex and heterogeneous disease characterized by the involvement of numerous cell types in its pathological processes, among which microglia play a pivotal role (Baecher-Allan et al., 2018). Emerging evidence indicates that activated microglia are not only present in active demyelinating lesions but also observed in normal-appearing white matter (Peferoen et al., 2015; Voet et al., 2019; Airas and Yong, 2022). Consistent with these findings, Zrzavy et al. demonstrated that microglia in the normal-appearing white matter of patients with MS exhibited significant activation, with their abundance correlating positively with disease duration compared to the control group (Zrzavy et al., 2017).

Microglia are usually activated into a proinflammatory phenotype during the acute phase of MS, typically characterized by enlarged cellular processes, enhanced migratory capacity, and increased phagocytic activity. These activated microglia upregulate the express of proinflammatory markers, including TMEM119, iNOS, major histocompatibility complex II (MHC II), CD40, CD45, CD68 and CD86 (Zrzavy et al., 2017). They actively participate in oxidative stress pathways by generating reactive nitrogen and oxygen species (ROS) (Mendiola et al., 2020) and secrete proinflammatory factors such as TNF-α, IL-1β, IL-6, IL-23 and IL-12. The above changes can activate complement system and recruit other immune cells within the CNS, ultimately contribute to demyelination, oligodendrocyte apoptosis, axonal degeneration and neuronal loss (van Olst et al., 2021; Lee et al., 2019). In contrast, during the chronic or remission phase of MS, anti-inflammatory microglia become predominant. These cells release anti-inflammatory factors such as IL-10, TGF-β, brain-derived neurotrophic factor (BDNF) and insulin-like growth factor 1 (IGF-1). Additionally, they facilitate tissue repair by recruiting oligodendrocyte precursor cells and promoting the differentiation of regulatory T cells (Tregs) (Michell-Robinson et al., 2015; Rigillo and Alboni, 2024).

Previous studies have demonstrated that Irisin could cross the blood-brain barrier and exert biological effects within the CNS (Islam et al., 2021; Phillips et al., 2014). In experimental autoimmune uveitis models, Irisin has been shown to modulate microglial polarization and mitigate disease severity by suppressing Th1 and Th17 cells responses, as well as reducing M1 microglial polarization through the HIF-1α signaling pathway (Wang MQ. et al., 2024). To further investigate the impact of Irisin on microglia in EAE, we examined microglial responses in EAE mice following Irisin intervention and validated these findings with BV2 microglial cells. The results revealed that Irisin not only ameliorated the clinical scores, reduced inflammatory infiltration and attenuated demyelination in spinal cord of EAE mice, but also significantly decreased microglia activation and downregulated the expression of iNOS. Consistent with the results of *in vivo* experiments, Irisin treatment markedly reduced inflammatory markers in BV2 cells, and suppressed the expression of proinflammatory phenotype-associated molecules, including iNOS. Furthermore, Irisin was found to target the microglial integrin αVβ5 receptor, promoting microglial reprogramming toward the M2 phenotype via STAT6 pathway and contributing to immune homeostasis (Wang JX. et al., 2024). Given the well-established role of microglia in EAE pathogenesis and the therapeutic potential of modulating microglial activation and polarization in EAE/MS treatment (Du et al., 2017; Wies Mancini et al., 2023), we propose that Irisin, with its ability to inhibit proinflammatory microglial polarization, holds significant promise as a therapeutic agent for EAE and MS.

The activation of NF-κB and STAT1 signaling pathways drives microglia toward a proinflammatory phenotype, resulting in cytotoxicity and inflammatory response. In contrast, the activation of STAT3 and STAT6 promotes microglial polarization toward an anti-inflammatory phenotype, thereby suppressing immune-mediated inflammatory reactions (Dheen et al., 2007). Previous studies have demonstrated that Irisin reduced IL-6 levels in adipocytes by downregulating the TLR4/MyD88/NF-κB pathway (Zhang YS. et al., 2024). In this study, we further investigated the underlying signaling mechanisms in both *in vivo* and *in vitro* models. Our findings indicated that Irisin inhibited the polarization of microglia toward a proinflammatory phenotype through modulation of the NF-κBp65 pathway. The NF-κB familycomprises five members: RelA (p65), RelB, c-Rel, NF-κB1 (p105/p50) and NF-κB2 (p100/p52). These proteins bind to promoter or enhancer regions to either induce or inhibit transcription (Zhang et al., 2017). The NF-κB pathway can be activated through both typical and atypical pathways. The typical pathway is triggered by TLRs and proinflammatory cytokines, leading to RelA activation, phosphorylation of IκBα, and nuclear translocation of heterodimers predominantly containing p65. In contrast, the atypical pathway is characterized by activation through lymphotoxin-β, CD40L, BAFF and RANKL, which depends on IKKα-mediated phosphorylation of p100, resulting in partial processing of p100 and the formation of the p52-RelB complex (Hayden and Ghosh, 2011; Oeckinghaus et al., 2011). Our research demonstrated that Irisin reduced microglial activation, a phenomenon potentially mediated through the NF-κBp65 pathway. These findings suggested that Irisin might exert its anti-inflammatory effects by modulating this critical signaling cascade in microglia.

## 5 Conclusion

**FIGURE 9:**
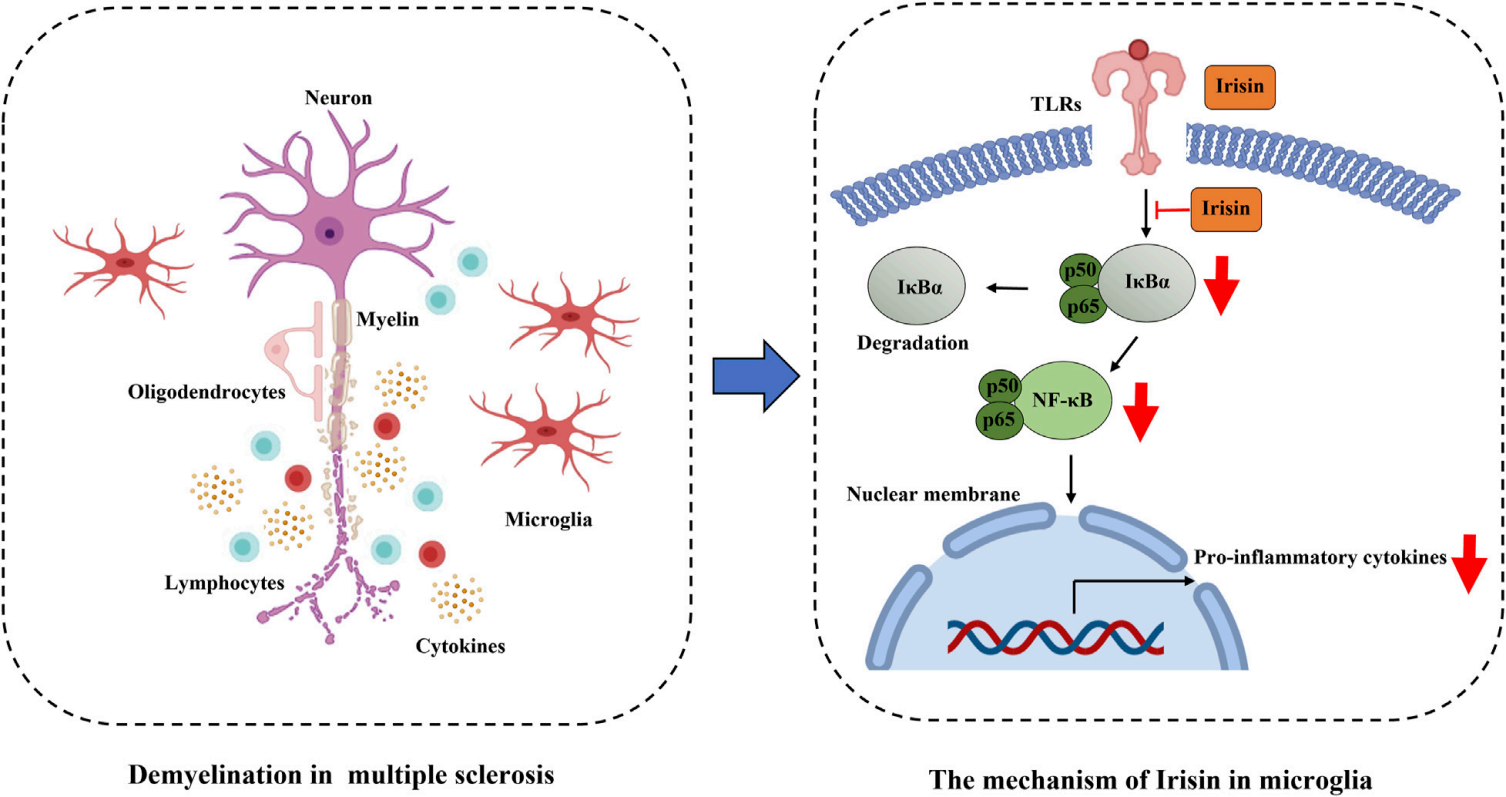
Irisin restrains microglia activation in EAE through the inhibition of NF-κBp65 pathway.

In summary, Irisin exerts a protective effect in EAE mice, significantly attenuating inflammatory response and reducing microglial activation in spinal cord. These beneficial effects may be mediated through the inhibition of NF-κBp65 pathway (Figure 9).

However, several limitations of this study should be acknowledged. Our investigation was limited to the analysis of pro-inflammatory markers and we did not assess anti-inflammatory markers in either the mouse model or BV2 cells. While we focused on the effects of Irisin on BV2 cells, future studies should validate these finding using bone marrow-derived cells or primary microglia to strengthen the translational relevance of our results.

## Data Availability

The original contributions presented in the study are included in the article/Supplementary Material, further inquiries can be directed to the corresponding author.
