# Supplementary figures and images for: Irisin restrains neuroinflammation in mouse experimental autoimmune encephalomyelitis via regulating microglia activation
Source: Front Pharmacol. 2025 Apr 29;16:1561939. doi: 10.3389/fphar.2025.1561939 (PMC12069398; doi:10.3389/fphar.2025.1561939)

**Fig.5 n=4**

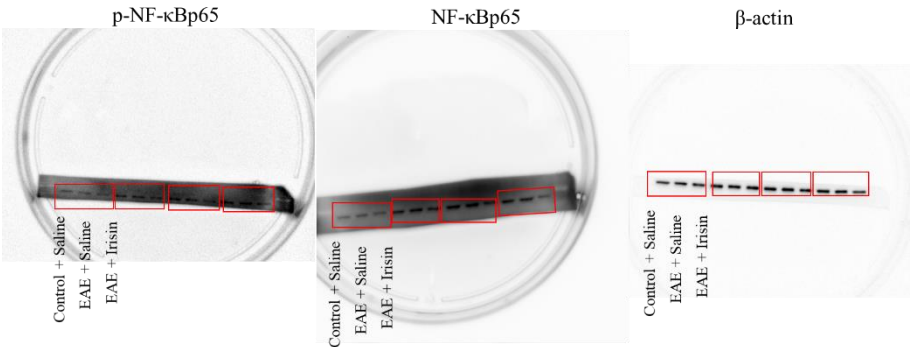

**Fig.8 n=4**

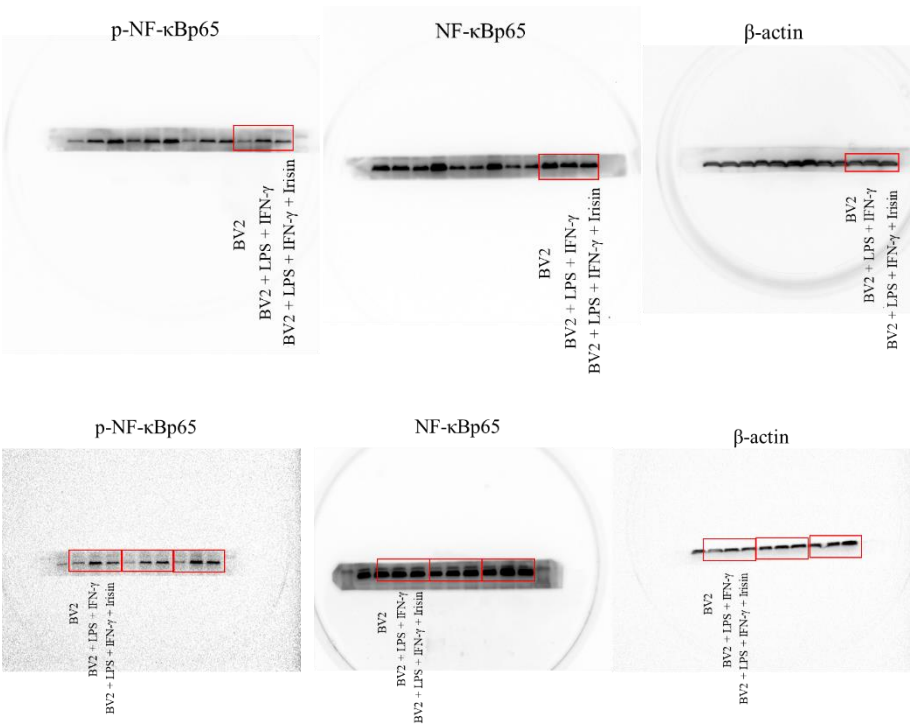

Supplement: Supplementary file 1 [file DataSheet1.pdf]
